# Immunization with a Thermostable Newcastle Disease Virus K148/08 Strain Originated from Wild Mallard Duck Confers Protection against Lethal Viscerotropic Velogenic Newcastle Disease Virus Infection in Chickens

**DOI:** 10.1371/journal.pone.0083161

**Published:** 2013-12-16

**Authors:** Seung-Hwan Jeong, Dong-Hun Lee, Byoung-Yoon Kim, Soo-Won Choi, Joong-Bok Lee, Seung-Yong Park, In-Soo Choi, Chang-Seon Song

**Affiliations:** Avian Disease Laboratory, College of Veterinary Medicine, Konkuk University, Seoul, Republic of Korea; University of Melbourne, Australia

## Abstract

Newcastle disease (ND) is one of the most devastating poultry infections because of its worldwide distribution and accompanying economical threat. In the present study, we characterized the ND virus (NDV) K148/08 strain from wild mallard duck, with regard to safety, thermostability, immunogenicity, and protective efficacy against velogenic ND viral infection. The NDV K148/08 strain offered enhanced immunogenicity and safety relative to commercially available vaccine strains. The NDV K148/08 strain was safe in 1-day-old SPF chicks after vaccination using a coarse or cabinet-type fine sprayer. We demonstrated that the NDV K148/08 strain elicited high levels of antibody responses and provided protective efficacy against lethal NDV challenge. In addition, the thermostability of the NDV K148/08 strain was as high as that of the thermostable V4 strain. Therefore, the NDV K148/08 strain may be useful to ensure NDV vaccine performance and effectiveness in developing countries, especially in remote areas without cold chains.

## Introduction

Newcastle disease (ND), which is defined as a reportable disease by the Office International des Epizooties (OIE), is a highly contagious viral disease of poultry and other bird species caused by virulent Newcastle disease virus (NDV) [1]. ND is one of the most devastating poultry infections because of its worldwide distribution and accompanying economical threat. NDVs have been categorized into lentogenic, mesogenic, and velogenic strains based on the disease severity in chickens [2]. Among these pathotypes, velogenic NDV causes severe economic losses to the poultry industry because it provokes severe neurological and respiratory signs as well as suboptimal egg production and egg quality [3,4]. For the prevention of ND, vaccination against ND using live vaccines is a common practice and even obligatory in many countries [4,5].

In vaccination methods, spray vaccination has been frequently used in the hatchery to immunize young birds against ND. The main advantage of spray vaccination is that a high number of birds can be immunized in a short period of time without being individually handled [6]. For spray vaccination of NDV, the vaccine elicits an adequate immune response with a minimal respiratory response [7]. Some vaccines including the V4 strain [8], VG/GA strain [9] and Ulster strain [10] are safe enough to apply to 1-day-old chicks via spraying. NDV strains originated from waterfowl have also been highlighted as potential vaccine candidates because of their natural attenuated characteristics [4,11].

In our previous study, during pathogen surveillance of wild birds from 2007 to 2009, NDVs were isolated from feces of wild waterfowl and genetically characterized [12]. In the present study, one of these strains, the NDV Anas platyrhynchos/Korea/K148/2008 (K148/08) strain, was selected as a live vaccine candidate and evaluated with regard to safety, thermostability, immunogenicity, and protective efficacy against velogenic ND viral infection.

## Materials and Methods

### Ethics Statement

All animal procedures performed in this study were reviewed, approved, and supervised by the Institutional Animal Care and Use Committee of Konkuk University.

### Characterization of NDV K148/08 strain

Nucleotide sequence of the fusion (F) gene was determined and used for characterization of the K148/08 strain of NDV. The F gene was amplified by RT-PCR as previously described [13]. Nucleotide sequence of the amplified fragment was determined with Sanger sequencing and submitted to GenBank under accession number KF724899. Phylogenetic analysis was performed with the MEGA 5 program using the neighbor-joining analyses with Kimura-2 parameter model. Nucleotide sequences of commercial vaccine strains [Ulster 2C (GenBank acc. AY562991), V4 (GenBank acc. AF217084), B1 (GenBank acc. M24695), and La Sota (GenBank acc. AY845400)] were included in this phylogenetic analysis. Statistical analysis of phylogenetic tree was determined using bootstrap analysis carried out on 1000 replicates.

The pathogenicity of NDV isolates was determined by standard assay methods for the intracerebral pathogenicity index (ICPI) in 1-day-old specific pathogen-free (SPF) chicks and for the mean death time (MDT) with the minimum lethal dose in 10-day-old SPF chicken embryonated hen eggs, as previously described [14].

 For the thermostability test, airtight sealed vials containing 1.0 ml of aqueous NDV K148/08 strain and a thermostable V4 strain were submerged into a water bath maintained at 56°C. The vials were incubated from 30 to 120 min, and after each 30 min interval, a vial was removed from the water bath and chilled rapidly in an ice-cold water bath to stop the heat treatment. Samples were then assayed for hemagglutination and infectivity activity. Hemagglutination assay was performed by standard micro-hemagglutination procedure using two fold serial dilutions of antigen in 25ul PBS in micro-titer plates. A 25 ul volume of the respective chicken erythrocyte suspension was applied to each well. These mixtures were incubated at room temperature for 40 min and the agglutination titers of the samples were determined as the reciprocal of the highest dilution giving 100% agglutination of 1% chicken RBC [15]. Infectivity titration was performed by embryonated egg inoculation and infectivity titers of the samples calculated by using the Reed–Muench method [16]. The experiments described above were repeated three times.

### Animals

SPF chicks were hatched from SPF embryonated chicken eggs (SPAFAS, Inc., USA) and raised in air-filtered bio-security isolation units (ThreeShine Co., Korea) with feed and water provided *ad libitum*. 

### Vaccines and virus

The K148/08 NDV vaccine candidate strain was isolated from the feces of wild mallard ducks in Korea [12]. Lentogenic or asymptomatic NDV vaccine viruses utilized in this study included a commercial vaccine strain derived from Hitchner B1 (Merial, France), VG/GA (Merial, France), and V4 (Bioproperties, Australia) strains. The viscerotropic velogenic NDV (vvNDV) strain Kr-005/00 was provided by the Animal, Plant and Fisheries Quarantine and Inspection Agency (Anyang, Korea) and used for the challenge study. The Kr-005/00 strain belonged to genotype VII and was isolated from laying chickens during an epizootic episode in Korea in 2000. All viruses were propagated using SPF chicken eggs, and the 50% egg infective dose (EID_50_) was determined. 

### Evaluation of safety profile

Two types of sprayer with different particle sizes were used in safety study. The most commonly used coarse sprayer had a 115 um particle size (Desvac^®^, Intervet, Netherland), and the cabinet-type fine sprayer had a smaller (50 um) particle size (SK-MO-Auto-3000^®^, Three-shine, Korea). Twenty-two 1 day-old SPF chicks per group were immunized with one dose (10^5.0^ EID_50_/bird) of the NDV K148/08 strain, NDV VG/GA strain, NDV V4 strain, or phosphate-buffered saline (PBS) using the coarse sprayer or cabinet sprayer. The birds were observed twice daily for clinical signs for 14 days. At 4, 7, 11, and 14 days after immunization, 4 chicks in each group were sacrificed and necropsied. The safety of the vaccine was evaluated by histological lesions. The histological lesions were scored as follows: 0, no obvious lesion; 1, mild; 2, moderate; and 3, severe.

### Evaluation of immunogenicity and protective efficacy

The protective efficacy of the NDV K148/08 strain was compared with that of commercially available vaccines. During experiment, birds were vaccinated via the intraocular route or using a cabinet-type fine sprayer and then challenged with virulent NDV. Non-vaccinated and challenged birds were used as control group. Twenty 1-day-old SPF chicks per group were inoculated 10^5.0^ EID_50_/0.1 ml of the NDV K148/08 strain, the NDV VG/GA strain, the NDV V4 strain, or PBS via the intraocular route. Ten 1-day-old SPF chicks per group were immunized with a dose of 10^6.0^ EID_50_ of the NDV K148/08 strain, NDV VG/GA strain, or PBS using a cabinet-type fine sprayer. Blood samples were collected on 14 days post immunization, and sera were isolated and subjected to hemagglutination inhibition (HI) assay. HI assay was done as previously described using a La Sota antigen [17]. Two weeks after immunization, all birds in each group were challenged with 10^5.0^ EID_50_/bird of vvNDV Kr-005 via the intranasal route. The birds were observed twice daily for clinical signs and death consistent with ND for 14 days to evaluate the protective efficacy of the vaccine against NDV. 

### Statistical analysis

Analysis of variance (ANOVA) with a Tukey–Kramer post-hoc test was performed for serum HI antibody titers. For comparison of protective efficacy between experimental groups, we employed the one-tailed Fisher’s exact test. Statistical significance was defined as a p value less than or equal to 0.05.

## Results

### Pathogenicity and thermostability

**Figure 1:**
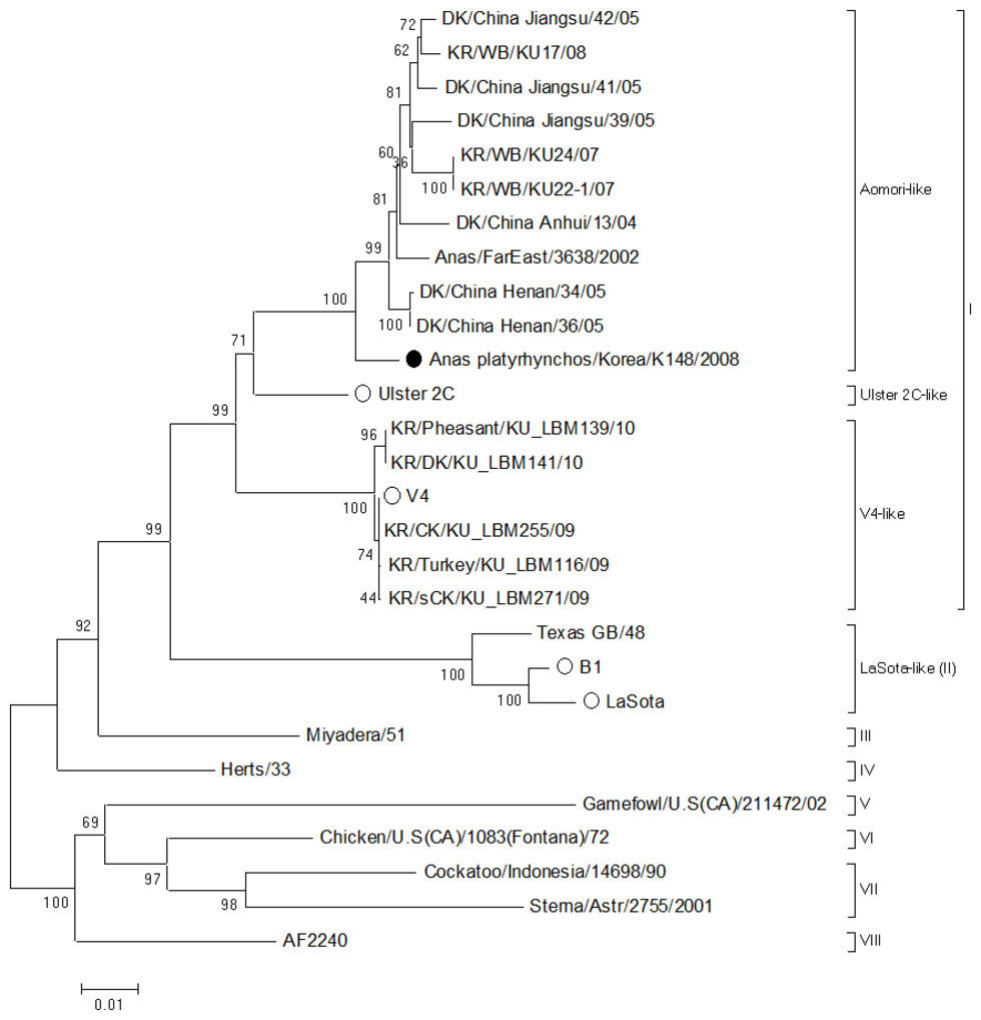
Neighbor-joining phylogenetic tree for the fusion gene of NDV isolates. Phylogenetic analysis was performed using the alignment of the first 1662 nucleotide sequence from the fusion gene coding region of NDV isolate. The K148/08 strain (●) was compared to commercial vaccine strains (○) obtained from the GenBank. The percentage of replicate trees in which the associated taxa clustered together in the bootstrap test (1000 replicates) were shown next to the branches.

For genetic analysis of the NDV K148/08 strain, we constructed a phylogenetic tree based on the first 1662 nucleotides of the F gene. The NDV K148/08 strain clustered together with the Aomori-like viruses in genotype I cluster (Figure 1). The amino acid residues of the F_0_ cleavage site motif were the same as those of commercial vaccine strains (Table 1). Standard pathogenicity tests (MDT and ICPI) were conducted to determine the virulence of the NDV K148/08 strain. As shown in Table 1, the NDV K148/08 strain was asymptomatic because the chicks showed a mean death time over 144 h and intracerebral pathogenicity index below 0.1. 

**Table 1:** Pathogenic index and molecular analysis of NDV K148/08 strain compared with commercially available NDV vaccine strains.

Vaccine strain	Pathogenic index		F_0_ cleavage site motif
	MDT ^a^	ICPI ^b^	
NDV K148/08	132 h	0.10	^112^ G-K-Q-G-R^116^-L^117^
NDV V4	156 h	0.00	^112^ G-K-Q-G-R^116^-L^117^
NDV Hitchner B1	114 h	0.20	^112^ G-K-Q-G-R^116^-L^117^
NDV VG/GA	144 h	0.00	^112^ G-K-Q-G-R^116^-L^117^

^a^ Mean death time

^b^ Intracerebral pathogenicity index.

The HA titers and embryonated egg infectivity of the NDV K148/08 strain after heat treatment were compared with those of the commercially available V4 thermostable vaccine strain and are shown in Table 2. HA titers and embryonated egg infectivity were maintained after heat treatment at 56°C for 120 min. These findings suggested that the ND K148/08 strain is a thermostable NDV strain.

**Table 2:** Hemagglutination activity and embryonated egg infectivity titer of NDV K148/08 strain after heat treatment at 56°C.

Virus	Parameter	Heat treatment time		
		0 min.	30 min.	60 min.
NDV K148/08	HA titer (Log_2_) ^a^	2^9^	2^9^	2^9^
	Infectivity titer ^b^	10^9.8^	10^9.5^	10^9.3^
NDV V4	HA titer (Log_2_)	2^10^	2^10^	2^10^
	Infectivity titer	10^9.3^	10^9.1^	10^9.1^

^a^ Hemagglutination assay titer

^b^ 50% embryonated egg infectious dose (EID_50_/ml)

### Safety in 1-day-old SPF chicks

Particle size of the sprayer is known to be an important factor for the safety and efficacy of live vaccines. In general, sprayers with a small particle size induce strong mucosal immunity, but also could induce more severe vaccine adverse responses such as respiratory signs and decreased weight gain. Safety study of the NDV K148/08 strain using coarse or fine sprayer was conducted in 1-day-old SPF chicks in comparison with commercially available vaccine strains (V4 and VG/GA strains). When the NDV K148/08 strain or commercial vaccine strains were immunized by a coarse sprayer, no clinical signs or histological lesions were observed in any bird throughout the period of the study (Data not shown). After immunization by a fine sprayer with the NDV K148/08 strain or commercial vaccine strains, no clinical signs were observed in any bird throughout the period of the study. However, as shown in Table 3, mild to moderate histological lesions in the respiratory tract were observed in all groups and the lesions disappeared by 11 dpi or 14 dpi in all groups. 

**Table 3:** Histopathological lesion score in day-old SPF chicks after vaccination using a fine sprayer with the NDV K148/08 strain or commercially available NDV vaccine strains.

Strain^a^	Histopathological lesion score **^*b*^**																
	4 dpi ^c^				7 dpi				11 dpi				14 dpi			
	Upper trachea	Middle trachea	Lower trachea	Lung	Upper trachea	Middle trachea	Lower trachea	Lung	Upper trachea	Middle trachea	Lower trachea	Lung	Upper trachea	Middle trachea	Lower trachea	Lung
K148/08	0	0	0	1	2	1	1	1	0	0	0	0	0	0	0	0
VG/GA	2	1	1	0	2	1.5	1	0	0	0	0	0	0	0	0	0`
V4	0	1	0	2	1	1	1	1	1	1	0	1	0	0	0	0

^a^ All birds in each group were immunized with a dose of 10^5.0^ EID_50_ using a fine sprayer (SK-MO-Auto-3000^®^ sprayer, droplet size 50 um)

^b^ 0: No obvious lesion, 1: Mild, 2: Moderate, 3: Severe

^c^ Day post inoculation.

### Protective efficacy and immunogenicity

The relative protective efficacy of the NDV K148/08 strain following vaccination via the intraocular route was investigated by challenging the chicks with virulent NDV. After challenge, the clinical signs and mortality were compared with those of non-vaccinated birds or birds that were vaccinated with the commercially available vaccines (V4, VG/GA, and B1 strains). As shown in table 4 and 5, All birds in the non-vaccinated and challenged groups died within 7 days post challenge (MDT = 4.0 days) after showing severe depression. In contrast, birds immunized with the NDV K148/08 strain were completely protected from the challenge infection. The immunogenicity of the NDV K148/08 strain following vaccination via the intraocular route or using a cabinet-type fine sprayer was determined by the HI test. There was a well-known correlation between HI titer and resistance to velogenic NDV, although low HI titers did not necessarily indicate absence of protection [18]. Based on our results, the HI titer of sera from chicks immunized with the K148/08 strain via the intraocular route was significantly higher than those from the non-vaccinated control group and groups immunized with the V4 or VG/GA strain, and as high as that from the group immunized with the B1 strain. The protection rates for chickens immunized with the NDV K148/08 and B1 strains were 100%, while the V4 and VG/GA protected 90% of chickens from the challenge infection. The mean HI titers of sera from chicks immunized with the K148/08 strain and VG/GA using a cabinet-type fine sprayer were 2^4.6^ and 2^4.0^, respectively. In these groups, both strains completely protected chickens from the challenge infection.

**Table 4:** Immunogenicity and protective efficacy in day-old SPF chicks vaccinated with the NDV K148/08 strain via the intraocular route and followed by challenge with genotype VII vvNDV.

Vaccine strain **^*a*^**	Number of birds	Mean HI titer **^*b*^** (S.D. **^*c*^**)	Mortality (%)
NDV K148/08	20	5.4**^*A*^** (1.24)	0/20 (0%)
NDV V4	20	3.2**^*B*^** (1.26)	2/20 (10%)
NDV VG/GA	20	3.9**^*B*^** (1.91)	2/20 (10%)
NDV B1	20	5.1**^*A*^** (0.90)	0/20 (0%)
PBS control	20	0.0**^*C*^** (0.00)	20/20 (100%)

^a^ All birds in each group were immunized with a dose of 10^5.0^EID_50_ through the eye-drop route.

^b^ Hemagglutination inhibition (HI) test was performed using sera from immunized birds at 14 dpi. Values represented with the same superscript letters for a mean HI titer are not significantly different (p < 0.05 by ANOVA with Tukey–Kramer post-test).

^c^ Standard deviation.

**Table 5:** Immunogenicity and protective efficacy in day-old SPF chicks vaccinated using a cabinet-type fine sprayer with the NDV K148/08 strain and followed by challenge with genotype VII vvNDV.

Vaccine strain ^a^	Number of birds	Mean HI titer ^b^ (S.D. **^*c*^**)	Mortality (%)
NDV K148/08	10	4.6 ^A^ (1.34)	0/10 (0%)
NDV VG/GA	10	4.0 ^A^ (1.41)	0/10 (0%)
PBS control	10	0.0 ^B^ (0.00)	10/10 (100%)

^a^ All birds in each group were immunized with a dose of 10^6.0^EID_50_ using a cabinet-type fine sprayer.

^b^Hemagglutination inhibition (HI) test was performed using sera from immunized birds at 14 dpi. Values represented with the same superscript letters for a mean HI titer are not significantly different (p < 0.001 by ANOVA with Tukey–Kramer post-test).

^c^Standard deviation.

## Discussion

Local application of attenuated live NDV vaccines via the spray or intraocular routes has been intensively applied to prevent ND [19]. Mild infection by the NDV vaccine can stimulate local immunity and prevent clinical NDV infection. However, as with any live attenuated vaccine administered via the respiratory tract at an early age, the NDV vaccine could damage the epithelial lining of the trachea. In addition, secondary bacterial infection could occur depending on the extent of the epithelial damage and cause poor growth performance. Based on our results, the NDV K148/08 strain induced mild histological lesions when a fine sprayer was used, but these effects were acceptable with respect to respiratory virus vaccine safety [20], and the level of damage was found to be less than or comparable to that of commercial vaccines. Therefore, the NDV K148/08 strain could be a new NDV vaccine candidate with a solid safety profile.

Thermostable vaccines can help to ensure vaccine potency in remote areas of the world with limited or no electricity available for cold chain refrigeration. In addition, thermostable vaccines hold promise for improving the application of vaccines by extending product shelf life, decreasing the cost of vaccine stockpiling, and easing the deployment of vaccines [21]. Previous studies on the thermostability of various strains of NDV indicated that most NDV strains lost their infectivity on exposure to temperatures of 50–55°C for 30 min [22]. Since then, several studies have shown that it is possible to select virus populations for heat resistance [23]. Based on our results, the NDV K148/08 strain was determined to be a thermostable vaccine strain because HA and infectivity were maintained after heat treatment at 56°C for 120 min. Thus, the NDV K148/08 strain could be used as thermostable NDV vaccine that can be taken into the field with minimum dependence on cold chains and refrigeration.

In the challenge study, NDV K148/08 and the commercial vaccine strains provided significantly higher protection than PBS. Post-immunization HI antibody titers were highest in the chicks receiving the NDV K148/08 strain, followed by intraocular vaccination. The titers in this group were significantly higher than those of negative control chicks and those of chicks that received the V4 or VG/GA strain. The HI titers observed in the present study are a good indicator of a protective immune response, as geometric mean HI titers ranging 2 log_2_ to 5 log_2_ gave been considered to provide clinical protection [24].

Generally, as the safety level of a live vaccine increases, the efficacy of the vaccine is likely to decrease. That is, a safer and more attenuated live vaccine is likely to be less efficacious. In the standard pathogenicity tests, the NDV K148/08 and commercial vaccine strains induced MDT over 132 hours and their ICPI were below 0.2. All strains examined in these tests were asymptomatic strains based on MDT and ICPI. Interestingly, although there was no significant difference in a standard pathogenicity and histopathology between tested strains, there was a tendency that the vaccine strains with higher MDT and lower ICPI induced higher HI titer and protective efficacy after vaccination via intraocular route. 

Currently, various live NDV vaccine strains are used worldwide to control the infection of NDV. For effective control of ND, NDV vaccines should offer enhanced immunogenicity and thermostability with a solid safety profile. Particularly, the biggest challenge to the development of a live NDV vaccine for 1 day-old chicks is to use a candidate virus strain as avirulent as possible to address the safety issue, while maintaining a substantial level of protective immune responses. In the present study, we demonstrated that the NDV K148/08 strain elicited high levels of antibody responses to the virus as shown by HI activity and provided protective efficacy against lethal NDV challenge. Importantly, The NDV K148/08 strain showed antibody responses that are comparable to the B1 strain and elicited a higher antibody response than V4 and VG/GA strains. Furthermore, the NDV K148/08 strain showed solid safety in 1 day-old chicks after vaccination with a fine sprayer that is comparable to V4 and VG/GA strains. Therefore, the NDV K148/08 strain has a potential as a novel live NDV vaccine candidate that is suitable for spray vaccination in the hatchery with highly immunogenic and safe characteristics. In addition, the thermostability of the NDV K148/08 strain was as high as that of the thermostable V4 strain. Therefore, the NDV K148/08 strain can help to ensure ND vaccine performance and effectiveness in developing countries, especially in remote areas.
